# Professional centred shared decision making: Patient decision aids *in practice *in primary care

**DOI:** 10.1186/1472-6963-8-5

**Published:** 2008-01-11

**Authors:** Duika Burges Watson, Richard G Thomson, Madeleine J Murtagh

**Affiliations:** 1 Institute of Health and Society, Newcastle University, 21 Claremont Place, Newcastle upon Tyne NE2 4AA, UK

## Abstract

**Background:**

Patient decision aids are increasingly regarded as important components of clinical practice that enable shared decision making (SDM) and evidence based patient choice. Despite broad acceptance of their value, there remains little evidence of their successful implementation in primary care settings.

**Methods:**

Health care practitioners from five general practice surgeries in northern England participated in focus group sessions around the themes of patient decision aids, patient and practitioner preferences and SDM. Participants included general practitioners (n = 19), practice nurses (n = 5) and auxiliary staff (n = 3). Transcripts were analysed using a framework approach.

**Results:**

We report a) practitioners' discussion of the current impetus towards sharing decisions and their perspectives on barriers to SDM, and b) the implementation of patient decision aids in practice and impediments such as lack of an evidence base and time available in consultations.

**Conclusion:**

We demonstrate two orientations to sharing decisions: practitioner-centred and patient-centred with the former predominating. We argue that it is necessary to rethink the changes required *in practice *for the implementation of SDM.

## Background

Engaging patients in clinical decision making has become a guiding ethical principle underpinning much contemporary and routine clinical practice [1-5]. Development of patient decision aids has been proposed as one component of clinical practice to enable evidence-based patient choice and shared decision making (SDM) [4]. Patient decision aids are designed to assist in decision making about healthcare by providing the best available evidence of the risks and benefits of particular therapeutic options in association with the elicitation and incorporation of patient values. When used in SDM it is anticipated that patients will be involved in the decision making process to the extent that they desire, and that decisions will be made in a partnership between patient and practitioner that acknowledges the rights and duties of all parties involved [6].

Despite broad acceptance of the use of patient decision aids in improving patient based outcomes and health, evidence for their successful implementation and use in extending SDM in routine practice remains scarce [7-10]. SDM is commonly contrasted with the paternalistic approach to decision making which is characterised by the expression, 'doctor knows best'. The shift towards SDM may then be seen to involve a philosophical reorientation that considers patient/practitioner relations differently, requiring (or perhaps producing) a new 'way of viewing the world' in which patients and practitioners share input into treatment decisions, and where patient values are taken into account [7]. Despite the wide-reaching implications of SDM for decision making in healthcare, there is little background information that addresses how and if patient decision aids are effectively incorporated by practitioners into their routine practice [8,11,12]. Others who have examined the practice of SDM have considered general practitioners[13], limiting their studies to practitioners who have an existing interest in and knowledge of SDM[10]. The current research project follows work within our research group developing and exploring patient and practitioner engagement and a computerised patient decision support tool for stroke prevention in atrial fibrillation [14-17]. Our key study objective for this research was to explore health care practitioners' perceptions and use of patient decision aids in routine clinical practice as a baseline study prior to an intervention involving the introduction of a suite of patient decision aids including the atrial fibrillation tool.

## Methods

Five general practice surgeries in northern England were invited to participate in the study. The selection of practices built on the recruitment in our earlier study. Within each of the participating practices information sheets were circulated and participants invited to attend a presentation. A one hour presentation was given by RGT to introduce the study, the concept of SDM and current developments concerning patient decision aids. The presentation included a brief overview of different approaches to decision making and gave examples of different patient decision aids and modes of implementation. It also introduced the work and resources of organisations involved in the development and use of patient decision aids such as the Ottawa Health Research Institute (OHRI) and International Patient Decision Aids Standards Collaboration (IPDAS) [18]. Participants were asked to reflect on the presentation, to consider the potential for using patient decision aids in their practice and to identify particular areas of clinical practice where a patient decision aid might be of value. After a two week period for reflection participants who had attended the presentation were invited to take part in focus groups to discuss further the topic of SDM and patient decision aids. The study received ethical approval from Sunderland LREC. All participants (Appendix 1) provided written consent to participate in the audio-recorded focus group. Focus groups (FG1-FG5) were conducted (by DBW) around the themes of SDM and patient decision aids in general practice, exploring potential for further development of SDM in the practice.

A framework approach was used (by DBW and MJM) in the analysis of transcripts [19]. The framework approach provides a procedural structure for qualitative research that enables a systematic approach to the data, whilst also allowing some flexibility in the interpretation. It has been identified as a suitable method for analysing data where the objectives of the research have been set in advance of the analysis, for example where particular themes are deduced as relevant to a research topic, or for policy-focused research [20]. The main objective of the focus group was to consider issues of relevance to SDM and patient decision aids in routine settings from the perspective of practitioners. A five stage process of analysis [19] was adopted involving: familiarisation with the data through reading and rereading the transcripts for recurrent themes; identification of a thematic framework based on the objectives of the research (Additional File 1); a process of indexing in which transcripts were annotated with codes derived from the thematic framework; summarising and synthesising this data into charts that use representative quotes to demonstrate themes (Tables 1, 2, 3, 4, 5, 6, 7). Some themes overlap with others. The analysis and interpretation is recognised to be the least well defined aspect of a framework approach. Taking a social constructionist standpoint enabled us to more clearly identify what was important to us in the transcripts: that is, the social construction of the objects of study (SDM and the use of decision aids in practice). During the analytic phase DBW and MJM employed the concept of reflexivity (cf. Griffin, 1995) which is described as a self-conscious awareness of the ways in which knowledge is produced by social relations of power and social position; race, class, gender amongst others. Moreover, we employed a variety of tactics to address concerns about validity in the research involving presenting representative quotes and undertaking a negative case analysis to look for disconfirming cases. Analysis was designed to generate themes of importance to SDM in routine practice and to use these to inform the introduction of SDM tools in routine practice within a subsequent intervention study.

## Results

### Sharing decisions

Different *practices *adopted approaches variably consistent with the principles of SDM. The transcripts from FG1 demonstrated the least familiarity with principles of SDM, and FG2 the most. In this section we demonstrate practitioner-centred (FG1 -Table 1) and patient-centred (FG2 -Table 2) approaches to sharing decisions. One GP in FG1 demonstrated a protective paternalistic approach to practitioner/patient relations in suggesting his role was to "explain what I think is best" to the patient and to hope the patient would share the decision by agreeing "that's fine." (Table 1:1). Discussing the topic of prescription of antibiotics for sore throat, the same GP argued that it was not appropriate, and even undesirable, to share information for decision making with the patient about this topic because while there may be limited benefit for some individuals, the adverse social consequences of using antibiotics were too great (Table 1:2). Practitioners in FG1 appeared concerned that patient decision aids might threaten their current roles. Two GPs associated patient decision aids with technological change and a declining role for humans, invoking dystopian images of "virtual GPs" (Table 1:3). Moreover, participants in FG1 appeared to have difficulty distinguishing between information giving and SDM. Asked how SDM was enabled in the practice one participant replied "I do share with patients', information. And the main source of information is my brain" (Table 1:4). Participants appeared reluctant to devolve decision making arguing that patients may not understand, may act irrationally with information given or may not act in the interests of public health (Table 1:5). The three practice nurses present in FG1 contributed very little to the discussion and when directly asked for their opinion on SDM by the researcher furnished short, single sentence answers. One, very recently employed, nurse demonstrated a more patient centred approach by suggesting that SDM involved "partnerships" with patients (Table 1:6).

**Table 1:** Representative quotes: practitioner centred practice (Focus Group 1)

1:1 Well I explain what I think is best ... and with a bit of luck they'll say "that's fine" and do it. (GP1 – Male)
1:2 There is one area where I don't particularly want decision aids or I don't want too much information [or] discussion, that's antibiotics because very few patients consider the public health implications of resistance. (GP1 – Male)
1:3 [If an interactive PDA introduced] you might lose doctors and clinicians altogether! Virtual GPs! (GP3 – Female)
1:4 I do share with patients' information and the main source of information is my brain... Shall we do A or shall we do B? (GP2 – Male)
1:5 Some patients are able to process the information that you give them very easily and other people might even be not able to read or the information that is given to them is very difficult to interpret. (GP2 – Male)
1:6 [SDM means] partnership in your decision making and in the care between you and patient. (Nurse practitioner – Female)

In contrast, FG2 appeared consistently to recognise and support a role for practitioners in facilitating patients' involvement in decision making (Table 2:1). In FG2 GPs still dominated focus group responses, but also actively encouraged those present (including a practice and health visitor) to participate in the discussion. Practitioners in FG2 were sympathetic and responsive to the importance of SDM in the consultation even though one GP described patient competencies negatively -suggesting that the basis of individual's decisions were "probably...irrational (Table 2:2)." Participants felt that their shared understanding and approach to SDM could be attributed in part to the adoption of a patient-centred culture in the practice, including regular involvement in training (Table 2:3). They were able to refer to key literature/authors on SDM (Table 2:4) and demonstrated they had applied the principles in practice by recording shared decisions when they occurred (Table 2:5). In FG4 participants expressed surprise that SDM was considered as something new (Table 2.6). When asked to reflect on SDM one GP suggested that most people wish to engage at some level, and that sometimes this engagement occurred outside the consultation as patients could (and sometimes did) reject what was offered as a treatment option (Table: 2.7). Despite recognising that decisions could be modified by patients because of the values they held, there was little mention of eliciting values as part of the consultation process itself, and not in any systematic way.

**Table 2:** Representative quotes: patient centred SDM practices (Focus groups 2 and 4)

2:1 At one end you've got doctors making decisions not giving patients any information or choice and at the other end you've got consumerism, essentially you just give them the choice and the price and all the other bits and pieces and they choose and shared decision making is some sort of negotiated pathway that involves both the doctors and the patients knowledge, opinions, experience. (GP2 – Male – FG2)
2:2 Its about empowerment...definitely empowerment... decision making ultimately impacts upon them. (Health Visitor – Female – FG4)
2:3 Information implies that decision making is relatively rationale where as of course most people make decisions probably on relatively irrational basis so it's actually acknowledging that, understanding that. (GP1 – Male – FG2)
2:4 We have communication skills regularly training with an outside trainer and its [SDM] one of the areas we've looked at. (GP2 – Female – FG2)
2:5 I went to a presentation by Glyn Elwyn who has done a lot on shared decision making and he went through some of the more sophisticated computer based tools. (GP2 – Male – FG2)
2:6 I've recorded it on the screen, you know. We came to a shared decision that it was appropriate not to have the medication. (GP2 – Female – FG2)
2:7 It seems an odd idea that we weren't doing it before, like somehow it's a new thing that wasn't going on before. (GP2 – Female – FG4)
2:8 ...you took the decision for them last time [but] they go away, they take a separate decision. (GP1 – Female – FG4)

Even those practices and participant practitioners sympathetic to SDM found the idea of sharing responsibility for decision making difficult 'in practice' (Table 3:1). First, some patients try to devolve responsibility for decisions to practitioners or expect them to take the decision (Table 3:2). Second, practitioners recognised that SDM often involved areas of uncertainty where neither the practitioner nor patient had sufficient information upon which to base a decision (Table 3:3). Third, some patients were reported to be more indecisive than others (Table 3:4) and practitioners found their role difficult because it involved assessing patients' decision making desires and abilities. Fourth, practitioners reported that it was difficult to remove themselves from the role of decision maker (Table 3:5). One GP suggested it was hard not to "push the decision", referring to the example of the older man who had shared a decision not to take preventive medication and who had subsequently had a stroke. She suggested that "a little bit of me thinks I should have been more forceful" [in making him take the medication] (Table 3:6). In such a situation the patient made a choice, but its legitimacy for the practitioner came into question with the perception of a 'wrong' outcome. Therefore, despite a recognition that "this generation don't like telling people what to do (Table 3:7)," there are powerful rhetorics at play in which responsibility is still seen to ultimately rest with the practitioner. While participants in FG2 considered it desirable to empower patients, "real shared decision making" involved a sometimes uncomfortable and difficult transition in practitioner roles. The importance of this observation is that some practitioners understood 'real' SDM as involving a fundamentally different power relationship between themselves and their patient, but one that challenged the largely unwritten social, ethical and emotional contract that has traditionally underscored patient/practitioner relations (Table 3:5, 3:6, 3:7). Practitioners across all focus groups suggested patients also failed to enact shared roles in decision-making. One respondent in FG2 suggested SDM felt more like "collusion" (Table 3:9)- a simulacrum of choice in which the role of the practitioner is to authorise the patient's decision through the rhetoric of SDM; and most often when it suited the practitioner: "I often find it easier to be involved in real shared decision making where I don't feel strongly either way (Table 3:9)."

**Table 3:** Representative quotes FG2: decision making complexities in patient-centred SDM

3:1 It's difficult to achieve equality but real genuine shared decision making is trying to do it from a position of equal basis, not necessarily equal knowledge but equal weight from both sides into making the decision and I think it's a bit of a challenge but very important. (GP2 – Female)
3:2 ...sometimes it is quite clear ... they want you to make the decision on their behalf... it's a very complicated part about decision making. (GP2 – Female)
3:3 You may be motivated to share the decision and either you don't have access to the information which would populate the decision boxes or there is no information about relative risk, benefit and all the rest... [it's] shared guess work. (GP3 – Male)
3:4 Its very tricky with indecisive people. (GP1 – Female)
3:5 Its actually very difficult for us to move back from the role of being the person who's really got the agenda, knows what should, or we feel should be done and not actually to sort of push the decision. (GP2 – Female)
3:6 I just heard yesterday he's had a stroke...a little bit of me thinks I should have been more forceful...you know we came to a shared decision that it was appropriate not to have the medication. (GP2 – Female)
3:7 I think our relationship has changed you know this generation we don't like to tell people what to do. (GP1 – Male)
3:8 I think we do, can collude with patients quite a lot. (GP1 – Male)
3:9 I often find it easier to be involved in real shared decision making where I don't feel strongly either way. (GP1 – Female)

Thus participants and practices varied in their awareness of the changed relationships inherent in the move towards patient-centred SDM. Practitioners' roles and relationships with patients were both institutionally driven and individually mediated. Where practices demonstrated commitment to principles of SDM and patient-centred practice, practitioners were more comfortable with the use of SDM language and principles and were able to recognise that SDM involved the shift of power implicit in the term. Despite facilitating SDM in practice, it was not clear that any practitioners in our focus groups felt completely comfortable with SDM or absolved from adopting decision making responsibility for patients.

#### Risk communication

The diversity of skills, methods and resources for sharing information with patients appeared to reflect *individual *as well as *practice based *differences. In FG2 sharing information about risk was viewed by some practitioners as "spurious" (Table 4:1) and unhelpful, whilst for others risks were useful "to grapple with (Table 4:2)." Participants in FG2 had also been involved in ongoing communication skills training. The expression of different opinions within the focus group and inclusion of all participants in the discussion demonstrated inclusiveness and openness to the views of others. In contrast, communication skills were not emphasised in FG1 or FG4. Rather, expectations about communication were based on common assumptions that participants would adhere to guidelines (Table 4:3). Different communication practices in the focus group may not reflect what happens in consultation. Nonetheless it is an indicator of preference for particular communication styles and suggests differences in how skills in communication are viewed as either common sense or learned. Different practices had invested more or less time and resources in communication and in pursuit of the particular goal of SDM as a "theory and an ideal (Table 4:4)."

**Table 4:** Representative quotes: means of communication

4:1 I very rarely get into detailed figures about risk and I think that to some extent they are, I mean part of that is philosophical, they are slightly spurious in the sense that they give this objectivity to it which just isn't there. (GP2 – Male – FG2)
4:2 I much prefer to have a figure to grapple with. (GP3 – Male – FG2)
4:3 We never discussed it [SDM] I don't think particularly. I would expect that everybody would be following the GMC guidelines: you know communication with patients. (GP1 – Male – FG1)
4:4 I think the culture here has been around patient centredness, as you know, a theory and an ideal. (GP2 – Male – FG2)

### Using patient decision aids

What was apparent across all focus groups was that practitioners had limited experience of patient decision aids *in hypothetical situations *or in *practice *to draw upon to describe how patient decision aids might be incorporated successfully. What was equally clear was that participants in those practices predisposed to a patient-centred approach and SDM were more likely to talk positively about using patient decision aids even when acknowledging the difficulties. Participants from more practitioner-centred practices were more likely to use the difficulties rhetorically to explain and justify their lack of use of patient decision aids and other SDM practices. When asked to reflect on the availability and use of patient decision aids in their practice, none had routinely used them. In FG3 two tools identified as patient decision aids used "regularly" were a risk/benefit table for using HRT (Hormone Replacement Therapy) and a Framingham derived computerised risk assessment for cardiovascular risk (by practice nurses and GPs). In both situations practitioners recognised that derivation of patient values were not incorporated in the use of the tools. Moreover, the presentation of risk was used to "reinforce what you are saying (Table 5:1)." What where considered patient decision aids, in practitioner-centred approaches, were useful if they produced decisions the practitioner was happy with (Table 5:2). The advantage of using a patient decision aid was viewed in terms of surveillance of the patient's health beyond the consultation. One GP suggested a blood pressure monitoring machine for home use "really helps me to make a decision" (Table 5:3) about whether or not to increase medication.

**Table 5:** Representative quotes: practitioner-centred use of decision aids

5:1 I use the coronary risk calculator and manipulated it by putting in a lower cholesterol value and showing people how their risk would come back... to reinforce what you are saying. (GP2 – Male – FG3)
5:2 I think that when you're using a decision making aid I think you can only do it if you're completely happy with the decision the patient will make from that. (GP2 – Male – FG1)
5:3 They come with a big list of all their blood pressures at different times and that really helps me to make a decision. (GP2 – Female – FG4)

#### Patient decision aids in practice

Some participants who had taken part in our earlier clinical trials of a computerised patient decision aid for atrial fibrillation [21-24] suggested the tools were "useful" (Table 6:1). In FG3 one participant suggested a Hormone Replacement Therapy decision tool had been particularly useful for improving practitioner knowledge (rather than facilitating SDM with the patient) (Table 6:2). Despite this, there was a perception across the practices that patient decision aids were not designed with 'real life' consultation pressures in mind. As one participant suggested of a computerised patient decision aid, it had been unfathomable to imagine how it could be incorporated into a 10 minute consultation (Table 6:3). Accessibility and lack of "faff" (fuss) appeared to be foremost in the minds of participants (Table 6:4). Nonetheless a number of difficulties in assessing how and when to use patient decision aids were identified. People have different conceptions of risk and risk communication may be confusing for both patients and practitioners (Table 6:5). Some patients are viewed as more needy of decisions than others and this neediness is difficult to assess (Table 6:6). Some patients may require different kinds of aids and support (Table 6:7). In other words, practitioners found it difficult to envisage using patient decision aids given the complexity of decision making, constraints of the consultation(s), the "faff," and the restrictions imposed by external priority setting (at both practice and policy levels). At the same time there appeared to be different motivations underlying the generally positive support and desire to develop SDM and use patient decision aids where possible. For one GP, patient decision aids were equated with simplified systems for information giving such that an "NHS bank of information and decision aids" that would reduce the current deficiencies of information retrieval and hand-outs (Table 6:8). Other participant practitioners were more reflexive of their desire to work towards "real shared decision making" and equality in the decision making process, but recognised they had limited skills or resources to do so (cf. Table 3:1). Whether in favour of using patient decision aids or viewing them as too much of a "faff," practitioners concerns predominantly centred around their own practice.

**Table 6:** Representative quotes: decision aids needs

6:1 We've actually got charts of smiley faces and I haven't used them, but when it was on IT [computer based] in the DARTS [Decision aids in Routine Treatment Study] I did find it useful. (GP2 – Male – FG2)
6:2 HRT risk errm, you know like how many people get breast cancer...that sort of thing and I use that regularly because it's quite easy to understand err, it helps me understand the figures! (GP2 – Female – FG3)
6:3 [They] went through some of the more sophisticated computer based tools but [it] seemed unfathomable to get them into a 10 minute consultation and actually he was in an internet site and using it in the consultation setting. (GP2 – Female – FG2)
6:4 It needs the decision aids to be there, it needs them to be readily accessible without faff and it needs experiential training of us. (GP2 – Male – FG2)
6:5 I get jumbled you know per 100,000 or 10,000 ...Your language is so subjective isn't it because it's how you interpret the risks. (GP2 – Female – FG2)
6:6 Some patients are able to process the information that you give them very easily and other people might even be not able to read or the information that is given to them is very difficult to interpret. (GP2 – Male – FG1)
6:7 [if it is a person] who can cope with the anxiety of not making a decision [in the consultation] it might be better to give him [sic] a decision making aid he could take away and work with. (GP2 – Female – FG2)
6:8 An NHS bank of internet information and decision aids would be absolutely a good aid because the downside of leaflets on your desk is a) keep an eye on the fact and that there still in date and b) if you want to get a bit of information from leaflets on different topics you would have no desk space left. (GP3 – Male – FG1)

#### Time

Time was a identified as a key constraint identified across all practices and is recognised in other studies [10,13,25]. Of all the potential barriers this best represents practitioner-centred thinking about PDAs and sharing decisions with patients. Relations between practitioners and patients were considered to be constrained by the brevity of the consultation and pressures associated with practice based targets. While it was observed that SDM may well have long term benefits in health outcomes, participants in FG4 shared the view that "selling" SDM to their practice would need to include some benefit that would, at the very least, not increase time spent with patients. The perception of SDM as time-consuming was used as a rhetorical device by a GP in FG1 to explain the lack of patient decision aids in that practice (Table 7:1). Time was treated as a fixed constant of the patient/practitioner interaction such that SDM could be cast, problematically, as an *additional element of the consultation *(Table 7:2 &7:3). Yet time, however much a hindrance, was not necessarily viewed as an immutable barrier. While one GP suggested "the big limiting factor is time;" he later observed that SDM might be thought of as "going on between consultations" (Table 7:4). The process of decision making was understood as on-going and not necessarily a 'one off' event limited to a one-off consultation: patient decision aids could have some utility as between-consultation tools. Other practitioners suggested patient decision aids could help make durable decisions that "save time in the future" (Table 7:3 &7:5). Other participants reported that receptionists could play a screening role identifying patients with particular health concerns, directing them to practitioners (including the practice nurse) with particular areas of clinical expertise – thus reducing the possibility that two consultations would be necessary; but also potentially creating a new space for SDM and patient decision aids outside the standard consultation.

**Table 7:** Representative quotes: saving time

7:1 You have to cut corners in everything and the amount of information you give and use of decision aids is one of the corners that you cut. (GP1 – Male – FG1)
7:2 I think it can be more time consuming as well to have a consultation [employing SDM]. (GP1 – Male – FG3)
7:3 I think probably long term it would save time but within your day to day practice you're very much working under a lot of pressure so it could be difficult to try and fit things in. (GP2 – Female – FG4)
7:4 Perhaps we should see SDM as a lot of that going on between consultations rather than during a consultation. (GP1 – Male – FG2)
7:5 More time making the correct decision ... would save time in the future. (GP1 – Female – FG4)

## Discussion

Our developmental study has addressed practitioner perspectives on SDM and patient decision aids in general practice settings in the UK through in depth analysis of focus group interviews. Our findings suggest that at both institutional and individual levels practitioners had different understandings and perceptions of their roles and relations in respect to patients that ranged from an implicit understanding and commitment to the principles of SDM that was patient-centred to a protective paternalism that was more practitioner-centred. Although almost one third of participants in focus groups were not GPs, discussions tended to be GP dominated or led. This therefore shaped our selection of representative quotes.

The results presented here reflect the preparatory nature of the study and our discussion and conclusions are limited by self-selected participation from within each practice. As we did not direct how participants engaged within the focus group, the representative quotes favour the views of the most dominant participants. While this may be viewed as a limitation, it may also be seen to reflect the organisational context of the general practice surgeries in which shared practices concerning decision making tend to be GP initiated and led. Regardless of these limitations, this study reveals important information on the present perspectives of general practice staff towards SDM and decision aids and the findings are critical to understanding how we might better address the challenges of supporting and implementing SDM in general practice, as well as indicating further research needs.

We identify two broad orientations towards SDM: patient-centred and practitioner-centred. In the first, practitioners recognise a changed relationship between practitioners and patients in *how *decisions get made. Patients' values about health are taken into consideration, and the patient/practitioner relationship is understood differently. In the second, SDM exists along a continuum of more and less paternalistic models: a philosophical reorientation of patient/practitioner roles is not evident in even the least paternalistic on this continuum. Rather, decisions may be shared in relation to issues where there is either uncertainty about the risks and benefits of particular treatment options, or in particular relation to prevention focused interventions. For practitioners historically charged with responsibility for a patient's welfare, the ability to devolve power is more difficult than might be suggested by the descriptions of SDM.

Rather than assuming that the traditional forms of evidence for patient decision aids and SDM are a sufficient base upon which to introduce them, in this study we considered a variety of health care practitioners' views about SDM and patient decision aids. In establishing a baseline of how practitioners themselves understand and see a role for SDM and patient decision aids we are thus able to reflect on the different understandings of SDM, different approaches to it, and different needs across practices and individuals. We propose that further research needs to address our findings that: SDM involves more than a change in practice and requires a new way of seeing the world; some practitioners are more successful than others in recognising such a shift but still find practice difficult; practitioners' views about their own and patient roles in decision making varied considerably across practices and individuals; practitioners/practices shared different understandings of where and when decision making gets done; practitioners were not equally skilled or knowledgeable about SDM; information needs and SDM were conflated; SDM terminology was sometimes used to describe relations of care in which practitioners maintained a largely traditional, paternalistic decision making role; patient decision aids were used as a means of surveillance of patients; SDM was sometimes perceived by practitioners as not 'real'; and practitioners had few examples on which to draw that demonstrate how patient decision aids can work in practice.

Several authors have recognised that SDM involves a philosophical reorientation away from earlier paternalistic models and new forms of thinking about patient-practitioner relations [7,26]. O'Flynn and Britten (2006) view such a reorientation in terms of the biomedical model, suggesting that the ability of practitioners to share decisions and devolve power to patients is, in reality, circumscribed because it is fundamentally in opposition to the practices through which they gain their professional identity. From this perspective it is not surprising that there are "low levels of SDM observed in practice" [see also 8] because practitioners are 'socialised' to a particular way of viewing practitioner/patient relations. O'Flynn and Britten (2006) surmise that practitioners need to do 'identity work', a reflexive re-examination of their role, if they are to achieve the goals of 'real' SDM. Elwyn (2004) also describes a shift in practitioner/patient relations. In his view however, the process of renegotiating patient/practitioner roles is a challenge but is also inevitable, given the growing recognition of uncertainties associated with decision making. In other words, for Elwyn (2004), new forms of patient/practitioner engagement are produced as an effect of managing uncertainties. The focus group findings we present here support the proposition that a transition in patient/practitioner relations is underway, but one that is recognised and advanced by some practitioners more than others. Reconfiguring health care practitioner roles in general suggests far broader changes in practice than introducing SDM for particular clinical conditions where there is uncertainty around treatment options or for prevention-focused interventions. What is less apparent to us is how much support practitioners and patients have in recognising and appreciating the difference between a new form of practitioner/patient relationship and the kinds of extended decision making roles that we have shown are sometimes described as SDM. Moreover, it is not clear what support is required to facilitate a more general shift towards patient-centred care.

Several recent studies have sought to address the apparent lacuna in understanding health care practitioners views on patient decision aids in clinical practice [9,12,13,27]. Findings of these studies tend to be conceptualised around ideas of the 'barriers' and 'facilitators' to operationalising SDM [13,25]. In these studies issues of time and lack of applicability for particular consultations are routinely represented as roadblocks to the successful implementation of SDM. Our approach differed in that we included a broader spectrum of professional expertise in primary care, including those already skilled in SDM and those with little previous knowledge. Moreover, in our analysis we considered the patient/practitioner interaction as well as the social and organisational context of decision making. We do not dispute the importance of others observations in helping to explain many of the difficulties practitioners associate with introducing patient decision aids into practice, or in helping develop solutions to some of the practical barriers and the identification of training needs of practitioners. However, the expectation that SDM occurs only within the consultation setting and between GPs and patients limits opportunities for introducing SDM in practice based settings. Moreover, such accounts do not take into consideration variations between institutions and individuals in how SDM is experienced or that the use of SDM terminology does not in itself guarantee that SDM is being adopted.

## Conclusion

Rethinking the changes required *in practice *for the implementation of SDM in primary care settings requires further examination. Current recommendations largely do not take account of the temporal, practical and other pressures in clinical practice. We support Elwyn's argument that viewing the consultation as an 'episodic didactic encounter rather than a longitudinal complex relationship' imposes limitations on how practitioners and patients can engage [7]. Across the focus groups different understandings of the consultation, and the role of SDM within that, produced different responses to the potential of SDM and patient decision aids. Where consultations were viewed as one off events requiring a decision, the potential for patient decision aids was viewed as limited and their design as requiring a time-saving element. Where the view of care was longer-term, involving more than one consultation and/or decision making as 'a dynamic process' in which patients were accorded a (new) role in SDM(FG2-GP1F – Table 2), the realm of possible uses for patient decision aids was greatly expanded. There is here scope for a greater attention to the concept of 'distributed decision making'; that is, understanding how 'decisions are distributed across time, courses of actions, people, situations and technologies' [24]. At the same time, the limitations of practice based targets and particular institutional organisations of referral that resulted in different levels of opportunity for the introduction of patient decision aids across practices need to be acknowledged.

### Practice implications

Our findings suggest that the implementation of patient decision aids in clinical practice should involve a more explicit recognition of the challenge of this approach and the implicit reordering of power that it may involve, however further research is required. First, evaluative frameworks and modes of delivering SDM tools into practice may need to address how different institutional settings and cultures modify the introduction of patient decision aids. Second, patient decision aids could be incorporated into routine practice beyond the confines of the consultation. Third, health care practitioners may need more supportive frameworks to enable them to do 'real shared decision making'; support that involves more than training in the methods of implementation and addresses the implications for health care practitioners that make devolving responsibility to patients immensely difficult.

## Competing interests

The author(s) declare that they have no competing interests.

## Authors' contributions

RGT led the presentation to practitioners, DBW organised and conducted the focus groups and participated in the data analysis sessions with MJM. All authors approved the final manuscript.

## Appendix 1

Focus group participation

FG1: GPs (3 male, 1 female); Nurse practitioners (3 female)

FG2: GPs (3 male, 2 female); Nurse practitioner (1 female); Health visitor (2 female)

FG3: GPs (3 female)

FG4: GPs (2 male, 2 female); Nurse practitioner (1 female); Health visitor (1 female)

FG5: GPs (2 female, 1 male)

## Pre-publication history

The pre-publication history for this paper can be accessed here:



## Supplementary Material

Additional file 1 A table of the thematic frameworks. Click here for file
