# Supplementary material for: Professional centred shared decision making: Patient decision aids in practice in primary care
Source: BMC Health Serv Res. 2008 Jan 11;8:5. doi: 10.1186/1472-6963-8-5 (PMC2248564; doi:10.1186/1472-6963-8-5)
Supplement: Additional file 1 — A table of the thematic frameworks. [file 1472-6963-8-5-S1.doc]

Additional File 1

Thematic Framework

| Main themes | Subtopics |
| --- | --- |
| SDM | Definitions involving theoretical discussion  Definitions employing practical descriptives |
| Practitioner roles in SDM | SDM threatens role  Power shift away from HCP  Decision making settings/beyond consultation  Deflecting personal responsibilities  Uncertainty of decisions  Legal and moral responsibilities |
| Practitioner /patient relations | Shifting views? responsibility for decisions  Sharing uncertainties  New relationships /new ways of relating |
| Patient and practitioner competencies | Differences in understanding  Patients fail to act appropriately  Patients modify decisions  Patient desires for involvement  Patient indecisiveness  Practitioner skills |
| Organisational settings | Skills development and aims  Capacities for SDM |
| PDAs | Definitions and descriptions of  Effect for decision making  Perceived limitations of  Possibilities and uses for |
